# Inferring Binding Energies from Selected Binding Sites

**DOI:** 10.1371/journal.pcbi.1000590

**Published:** 2009-12-04

**Authors:** Yue Zhao, David Granas, Gary D. Stormo

**Affiliations:** Department of Genetics, Washington University School of Medicine, St. Louis, Missouri, United States of America; T.U.Denmark - Center for Biological Sequence Analysis, Denmark

## Abstract

We employ a biophysical model that accounts for the non-linear relationship between binding energy and the statistics of selected binding sites. The model includes the chemical potential of the transcription factor, non-specific binding affinity of the protein for DNA, as well as sequence-specific parameters that may include non-independent contributions of bases to the interaction. We obtain maximum likelihood estimates for all of the parameters and compare the results to standard probabilistic methods of parameter estimation. On simulated data, where the true energy model is known and samples are generated with a variety of parameter values, we show that our method returns much more accurate estimates of the true parameters and much better predictions of the selected binding site distributions. We also introduce a new high-throughput SELEX (HT-SELEX) procedure to determine the binding specificity of a transcription factor in which the initial randomized library and the selected sites are sequenced with next generation methods that return hundreds of thousands of sites. We show that after a single round of selection our method can estimate binding parameters that give very good fits to the selected site distributions, much better than standard motif identification algorithms.

## Author Summary

The DNA binding sites of transcription factors that control gene expression are often predicted based on a collection of known or selected binding sites. The most commonly used methods for inferring the binding site pattern, or sequence motif, assume that the sites are selected in proportion to their affinity for the transcription factor, ignoring the effect of the transcription factor concentration. We have developed a new maximum likelihood approach, in a program called BEEML, that directly takes into account the transcription factor concentration as well as non-specific contributions to the binding affinity, and we show in simulation studies that it gives a much more accurate model of the transcription factor binding sites than previous methods. We also develop a new method for extracting binding sites for a transcription factor from a random pool of DNA sequences, called high-throughput SELEX (HT-SELEX), and we show that after a single round of selection BEEML can obtain an accurate model of the transcription factor binding sites.

## Introduction

Sequence-specific DNA binding proteins, including many transcription factors (TFs), are a critical component of transcriptional regulatory networks. Knowing their quantitative specificity, both the preferred binding sites and the relative binding affinity to different sites, can facilitate the understanding of gene expression patterns and how they are affected by altered cell states and variations in the genome sequences. A variety of methods are used to estimate the quantitative specificity of DNA-binding proteins, some of them direct experimental measurements of individual sequences or a few sequences at a time [1]–[5]. There are also new high-throughput methods that return quantitative, or at least semi-quantitative, binding affinities for many more sequences at a time [6]–[8]. Other methods are based on statistical analyses of example binding sites where the most commonly used methods are based on a probabilistic model of binding in which the frequencies of the observed bases at each position in the binding sites are used to estimate the probabilities of the complete sites being bound [9]. That approach misses the non-linear relationship between binding energy and binding probability that is especially critical for sites that have high occupancy, which can include the most important functional sites [10],[11]. In this paper we describe a method to obtain maximum likelihood estimates of binding energies based on a biophysical model of protein-DNA interactions and experimental data from high-throughput sequencing of *in vitro* selected binding sites.

### Biophysical model and site statistics

The bimolecular interaction between a DNA binding protein, TF, and a particular DNA binding sequence, *S_i_*, is governed by two rate constants, *k_on_* for the formation of the complex, and *k_off_* for the dissociation rate:
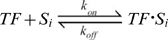



The equilibrium binding constant of the TF to the site *S_i_* is:
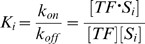
where concentrations are indicated by the brackets. At a specific instant, *S_i_* can be in two possible states, bound or free, indicated by *s = 1* or *s = 0*, respectively. The probability of TF binding to sequence *S_i_* is:
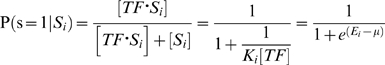
(1)where 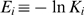 is the standard free energy of binding (often referred to as 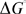), in units of RT (R is the gas constant and T the temperature in degrees Kelvin) and 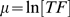 is the chemical potential [10],[11]. We expect that the binding energy can be decomposed into two, or more, modes of binding [11]. In the following analysis we assume two modes, non-specific binding that is independent of the sequence [11],[12], and specific binding that varies with different sequences such that
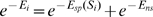
(2)


The specific binding component,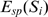, could be a complex function of the sequence, even itself being composed of multiple modes of binding. But for most of the examples in this paper we assume a simple additive energy function that can be represented as a position weight matrix (PWM) [9]. This model requires an energy contribution, 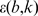, for each base, *b*, at position, *k*, in the binding site such that
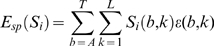
(3)where 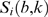 is an indicator variable with 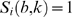 if base *b* occurs at position *k* of 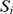, and 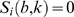 otherwise. The model can be easily extended to include energy contributions from combinations of bases, such as di-nucleotides or higher-orders [13]–[17].

Equation (1) is derived by considering a simple experiment where only a single sequence, 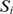, is available for binding, but holds true in the more general case where there are many different sequences all competing for binding to the TF. However, the interpretation 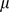 is different between the simple and general case. In the simple experiment, TF not bound to 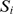 are simply free in solution, so 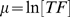. In the general case, TF not bound to 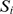 could be bound to any of the other available sequences, so *μ* corresponds to a free energy for the collection of all of the states with the TF not bound to 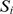. We present an alternative derivation of equation (1) to further illustrate this point. Consider that at any given time a particular sequence, 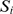, can be in one of three possible states: bound to the TF in the specific binding mode (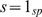; bound to the TF in the non-specific binding mode 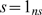; unbound by the TF 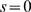. At equilibrium the probability of being in each state is determined by the energy of that state according to the Boltzmann distribution:
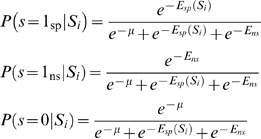
(4)


The overall probability of the sequence being bound (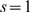) is the sum of the specific and non-specific binding probabilities. Using equations (2) and (4):
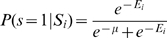
(5)which is equivalent to equation (1) but now for the general case of many sequences competing for the same pool of TF.

The experiment we model is a binding reaction with a pool of TF molecules and a large pool of different sequences, *S_i_* (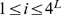 for the list of all possible sequences of length *L*), and with each sequence in proportion 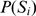 which can be determined with high-throughput sequencing. At equilibrium the TF molecules are extracted from the reaction along with the DNA sequences bound to them. The bound DNA sequences are subjected to high-throughput sequencing to obtain a large collection of binding sites, with the proportion of each sequence being 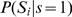, which is related to equation (5) using Bayes' rule:
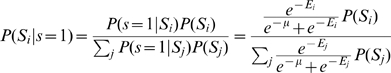
(6)


As pointed out by Djordjevic *et al* [10], the traditional log-odds method is appropriate only for a special case of equation (5) in which the TF is at very low concentration (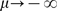) making the denominator of equation (5) a constant (independent of 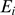) so that the probabilities of binding to each sequence, equation (6), are in direct proportion to their binding affinities.

Given a large enough sample of binding sites this experimental procedure could provide good estimates of the binding free energy for each sequence in the initial pool. However, for typical lengths *L* and typical differences in binding energy this would require an extremely large number of binding sites, more than available even from current high-throughput sequencing methods. By employing a model for the binding energy, such as equation (3), we can infer binding energies for sequences with limited or inaccurate measurements. Furthermore, having a model for the sequence dependence of the binding energy, instead of just a list of binding energies to different sequences, can be useful in understanding the physical interaction of the protein with the DNA and can facilitate the prediction of changes in binding energies for variant proteins [18].

Equation (1) was used by Djordjevic *et al* [10] as the starting point in the development of their QPMEME method. However, QPMEME makes the additional assumption that all observed sequences are bound with probability close to 1 (the zero temperature approximation) which prevents it from making use of the quantitative data generated by the HT-SELEX method in which many of the observed sites after one round of selection have low, even non-specific, binding affinity. A direct comparison with our approach is not possible because QPMEME fails to find a solution on datasets containing many low affinity sequences. An equivalent model was used in the TRAP algorithm by Roider *et al* [19] in the context of estimating total occupancy in ChIP-chip experiments. In TRAP the specific energy model (PWM) is assumed to be known and 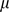 is estimated from the data, whereas we attempt to learn both the energy model and 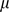 simultaneously.

This completes the description of the model. By substituting equation (3) into equation (2), and that into equation (6), we obtain the relationship between the statistics of observed binding sites, 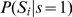, and the binding energy of each sequence, 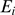. The set of unknown parameters, 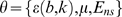, are estimated using a maximum likelihood approach (see Methods).

## Methods

### Maximum likelihood parameter estimation

Given a collection of N bound sequences, we model the relationship between 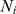, the number of occurrences of each sequence 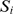 in this collection, and 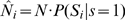, the number of occurrences of 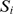 predicted by the model, as:
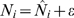
where 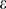 is a measurement error due to sequencing error as well as the stochastic nature of the sampling. For simplicity we assume 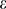 is a zero-mean Gaussian random variable with standard deviation 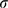, although other error models are possible [20]. For any set of parameters 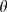 the probability of the data is
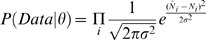
(7)


Maximizing the likelihood function (7) with respect to 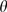 is equivalent to minimizing the negative log-likelihood function. Dropping the terms that do not depend on 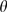, we have the objective function:
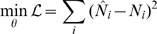
(8)


This is a non-linear parameter estimation problem and we minimize 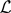 using the Levenberg-Marquardt algorithm implemented in minpack [21].

A practical issue is the calculation of the denominator of equation (6), the partition function. For longer values of *L* the naïve approach of enumerating over all sequences becomes too computationally expensive. We deal with that situation by rewriting equation (6) as
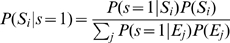
(9)where 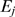 is a particular energy level. Instead of summing over all *4^L^* sequences, equation (9) allows us to sum over a user-defined number of energy levels (default is 16384) with some loss of accuracy due to the discretization. This does not solve the problem by itself, merely shifts it from enumerating all sequences to the calculation of the energy distribution 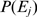. The naïve method of calculating 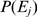 is to compute binding energy for all sequences and 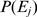 is simply the fraction of sequences having energy level 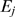. A more efficient method is possible under the PWM energy model by taking advantage of its Markovian nature. In this method, each position in the PWM is represented by a probability generating function, unequal priors are accounted for by the coefficients of the generating function. The distribution of energies defined by the entire PWM is obtained by multiplying the generating functions for each position [22]. This polynomial multiplication can be performed efficiently with a Fast Fourier Transform (FFT) [23]. By default, FFT approximation is used for binding sites of 10 and longer. This approach is implemented in an R [24] program called BEEML (Binding Energy Estimates using Maximum Likelihood) and is available from the authors on request.

### Simulated data

**Figure 1:**
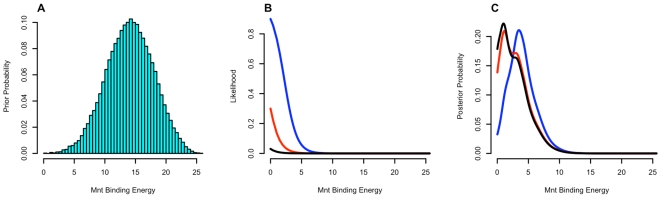
Effect of Mu on binding probabilities. (A) Prior distribution of binding energy for Mnt half-site [3],[27], with equiprobable background frequency. (B) Binding probability as function of binding energy, according to equation (1). Colors correspond to values of 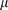, Black: 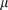 = −3.48, Red: 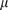 = −0.85, Blue: 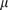 = 2.2. These values were chosen such that binding probabilities of the consensus sequence are 0.03, 0.3 and 0.9, respectively. No non-specific binding energy is used. (C) Posterior distribution of binding energy.

We use the half-site of the Mnt protein to test the method. Mnt is a repressor from phage P22 for which the binding affinity to all single base variants of the preferred binding sequence have been measured experimentally [3],[25]. We use the convention that the preferred base in each position is assigned an energy of 0 and all other values are positive and represent the difference in binding free energy, 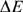 (or 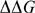 relative to the preferred base, attributed to each of the other bases [26]. Figure 1A shows the distribution of binding energies over all 7-long sequences for the half-site energy matrix of Mnt [3],[27]. Figure 1B plots the probability of drawing a sequence with a specific energy, from equation (1), for three different values of *μ* in which the probability of the binding to the preferred sequence (with 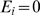) is 0.03, 0.3 and 0.9. Figure 1C shows the posterior distribution of binding energies which is the normalized product of the plots in Figures 1A and 1B, as in equation (9). This plot does not use a non-specific binding energy but that is employed in some of the simulations described later. Including 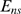 has the effect of essentially truncating the distribution at that point and all of probability density that would have been higher accumulates at 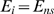. Using various values of 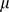 and 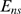 and setting 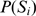 to a constant (equiprobable background distribution) 100,000 sites were drawn for each simulation according to equation (9).

### Quantitative binding data

We also used BEEML to analyze the binding data for the human transcription factor MaxA. Binding affinities to all possible 4-long half sites, in the context of the preferred GTG for the other half-site, were determined experimentally by the MITOMI method [8]. In this case we do not have a known binding energy model but rather the binding affinities to a large collection of binding sites.

### HT-SELEX data

The zinc-finger protein Zif268, fused to Glutathione-S-Transferase (GST), was previously purified from an E. coli expression system for use in SELEX experiments [28]. We augment the standard SELEX approach by incorporating Illumina sequencing of both the initial library and the selected sites and show that this high-throughput SELEX (HT-SELEX) procedure can obtain accurate binding energy models from only a single round of selection. The GST-tagged DNA-binding domain of Zif268 was mixed with 100-fold molar excess of a 56bp dsDNA template containing a 10 bp randomized region and incubated at room temperature for 1 hour in 1× reaction buffer (30mM Tris-HCl pH 8, 50mM NaCl, 0.1mg/ml BSA, 3mM DTT, 20uM ZnSO4, salmon-sperm DNA 25ug/ml). Glutathione sepharose resin was equilibrated in 1× reaction buffer and then added to the DNA-protein mixture for another hour of incubation. The mixture was added to a polypropelene column and unbound DNA was washed off with 5 ml of 1× reaction buffer added dropwise. The sepharose resin containing bound protein-DNA complexes was added directly to a PCR mix. DNA was amplified for 25 rounds of PCR and ethanol precipitated. The resulting DNA template was extended with an 111bp DNA fragment from pNEB193 to generate an optimal product length for Illumina sequencing. Both the initial library of randomized DNA, and the library of selected binding sites was subjected to Illumina sequencing and over 200,000 sites were obtained from each library.

## Results

### Simulation data

**Figure 2:**
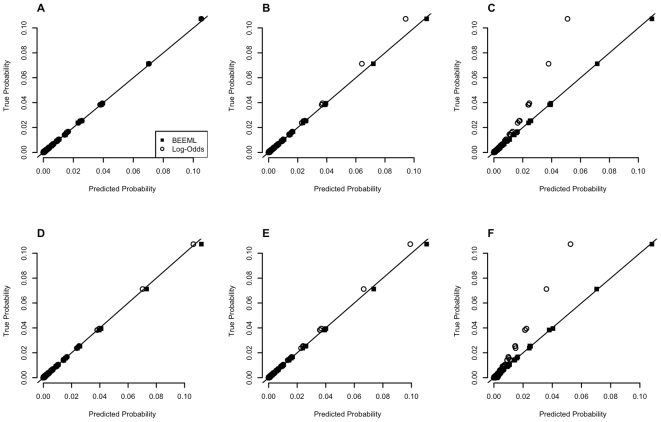
Examples of Simulation Results. Top Panel (A–C): Effects of 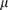. Non-specific energy was set to 30 so as to have negligible effect on binding. (A) 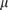 = −3.48 (B) 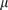 = −0.85 (C) 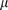 = 2.2. Bottom Panel (D–F): Effects of 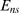 at low concentration limit. 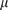 was set to −100. (D) 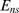 = 13.82 (E) 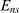 = 11.51 (F) 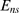 = 9.21. These values were chosen such that the relative 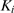 of consensus sequence to non-specific binding is (D) 1,000,000 (E) 100,000 (F) 10,000.

Figure 2 shows the performance of BEEML at predicting the true binding probabilities in the Mnt simulations for several different values of 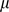 (Figure 2A–C) and 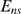 (Figure 2D–F). Each graph shows the true probabilities for all sequences and the predicted probabilities obtained by BEEML and also using a standard log-odds approach where the probabilities of each base at each position are taken directly from the observed sites. When 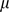 is low both methods give quite accurate predictions of binding probabilities. But at higher values of 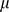, when the highest affinity sites approach saturation, the log-odds method is much worse at predicting the binding probabilities. Even when the preferred site is bound with p = 0.3 (Figure 2B), which is less than half saturated, there is a substantial difference in accuracy of predicted binding probability. At p = 0.9 for the preferred site (Figure 2C), the predictions from the log-odds method are wrong by about a factor of 2, whereas the BEEML predictions are very accurate. Many TF binding sites *in vivo* are likely to function at near saturation, especially those regulated by repressors, and inaccurate models for the binding probabilities can lead to very large increases in the number of false positive predictions of regulatory sites [10],[19],[27].

Similar results are obtained for variations of 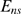. When 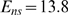 (which corresponds to a 10^6^-fold ratio of non-specific binding affinity compared to the preferred binding site, Figure 2D) both methods give accurate predictions of binding probabilities. But when it is reduced to 11.5 (ratio of 10^5^, Figure 2E) the log-odds method is less accurate, and when it is reduced to 9.2 (ratio of 10^4^, Figure 2F) the log-odds predictions are wrong by about a factor of 2, whereas the BEEML predictions are still very accurate because it can specifically account for that parameter whereas the log-odds method cannot.

### Quantitative binding data

**Figure 3:**
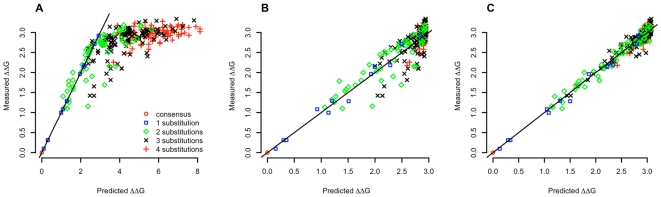
Re-analysis of Maerkl & Quake data. (A) Fit of point-estimate of binding energy as done in Maerkl & Quake paper (B) BEEML fit with PWM energy model and non-specific energy parameter (C) BEEML fit with position specific di-nucleotide energy model and non-specific energy parameter. (Note that in a previous analysis of this data [28] there was an error in equation (2), and equation (2) from this paper is the correct model.)

Figure 3 shows similar results for the analysis of the MaxA binding affinity data. Figure 3A comes directly from quantitative binding data where the measured binding energies are plotted versus the predictions assuming that multi-position variants show the additive energy changes of the individual base changes [8],[28]. As the authors point out, this additive assumption is not very accurate and the fit between the observed and predicted binding energies has only r^2^ = 0.57. Figure 3B plots the predictions from BEEML which estimates 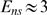 (much lower than in the simulations described above) and finds the best overall additive parameters, which together lead to an improved r^2^ = 0.84. Figure 3C goes one step further and estimates maximum likelihood parameters for nearest neighbor contributions to the binding energy. Using these adjacent di-nucleotide parameters increases the fit to r^2^ = 0.96, which is essentially within the measurement error.

### HT-SELEX data

**Figure 4:**
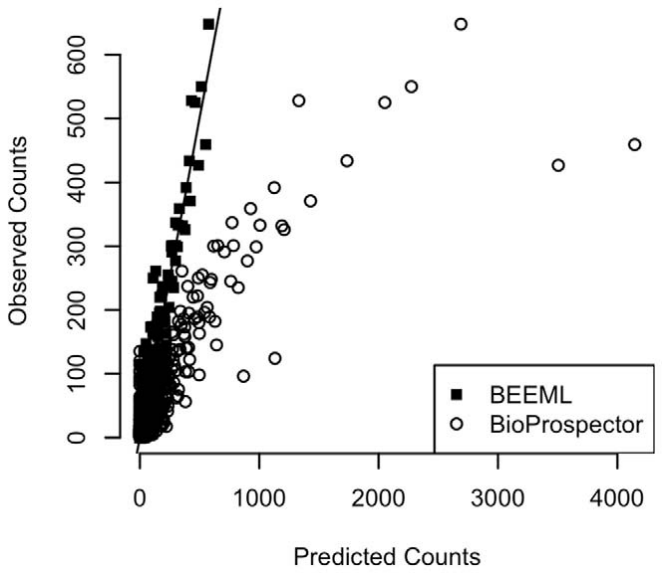
Fit of BEEML and BioProspector model to SELEX data.

The sequencing of the initial library showed a small bias in the composition on the synthetic strand: A = 24.5%; C = 21.0%; G = 27.2%; T = 27.4%. We estimate the prior probabilities of sequences, 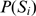, based on the mono-nucleotide composition. It is possible to measure 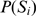 directly by sequencing the initial library more deeply, but in these experiments we only obtained about 200,000 sequences from each library, too few to estimate the frequencies of all 4^10^ (>10^6^) 10-mers. Since no significant higher-order biases were observed we expect that the frequencies of all 10-mers in the initial library are well approximated based on the mono-nucleotide composition. An initial BEEML model based on all of the selected binding sites was used to determine the most likely orientation of each site and whether it was entirely within the 10bp randomized region or overlapped the fixed sequences. Sites that were determined to overlap the fixed regions were eliminated from further analysis and the remaining sequences were reanalyzed by BEEML. As expected, because of the slight compositional bias and the G-rich consensus for zif268 (GCGTGGGCGT [29]), more sites were selected in the “top” orientation than in the reverse. When computing the likelihood we sum over binding in both orientations. Figure 4 shows the observed and predicted counts for all of the sequences in the selected set based on the BEEML model and also for a model obtained using BioProspector [30], a motif discovery program designed for this type of data. From the total of 259,704 sites, BioProspector built a model based on only 28,046 (10.8%) sites, but obtained a model that is similar to the known zif268 binding model. While BioProspector identifies the known consensus sequence and the PWM it finds is similar to previously published ones for zif268 [29], its quantitative predictions are much worse than those from the BEEML model (r^2^ = 0.74 for BioProspector, r^2^ = 0.92 for BEEML). Not only are the non-specific and low affinity sites, which are the majority after only a single round of selection, better predicted by BEEML, but the high affinity, near-consensus sites are predicted much more accurately and with very little scatter compared to the BioProspector predictions. BEEML also returns estimates of 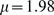 and 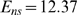. The predicted non-specific binding ratio of 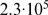-fold less than to the consensus sequence is in the range typical for many TFs. The estimate of 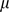 predicts that the consensus sites should be about 88% bound which is reasonable because, even though DNA is in 100-fold excess over protein in these experiments, most of the DNA sequences will have only non-specific affinity. This makes the experiment similar to the simulation depicted in Figure 2C and highlights the importance of the biophysical model instead of the log-odds approach. Because we are estimating only 32 parameters (30 for the PWM, and 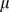 and 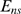) and have >10^5^ binding sites, we do not expect any over-fitting but to verify that is the case we performed a 10-fold cross-validation where we determined the parameters based on a random sample of 90% of the sequences and measured the fit to the remaining 10%. Indeed, we find that r^2^ = 0.90±0.05 on those samples.

## Discussion

Probabilistic models for binding site recognition, such as the fairly standard log-odds method, are popular because of their simplicity, intuitive appeal and because they can be easily implemented in motif discovery algorithms [9]. But they suffer from over-simplification of the underlying model, not just the typical additivity assumption which is known to be an approximation, a good one sometimes and other times not [31], but also because it ignores the non-linear relationship between binding energy and site statistics which is especially pronounced when high affinity binding sites approach saturation. The biophysical model [10],[11] captures the non-linear dependence of the binding probability on the energy and can easily incorporate multiple modes of binding, even beyond the specific and non-specific contributions that we employed in this study. It can easily incorporate non-additive, or higher order, contributions of the sequence to the binding energy, as we demonstrated on the MaxA data. Although not described in this work, the model can be further extended to include cooperativity, both positive and negative, between multiple factors binding to nearby, or even overlapping, sites [32].

Djordjevic *et al* [10] developed a quadratic programming (QP) method to estimate binding energy parameters from example binding sites and demonstrated that the resulting model could make many fewer false positive predictions on genome sequences. We have compared the accuracy of the QP approach to a standard log-odds method and also to the MATCH program [33] on many different simulated datasets selected under a variety of different sampling constraints, and showed that in many cases it could produce significantly more accurate models and greatly reduce the false positive rate [27]. But QP is still limited in the kinds of data for which it works well. It assumes a “zero temperature” limit for the binding probability so that sites either bind or not, rather than have a specific probability of binding. It functions like a support vector machine trained on only positive examples and is very sensitive to any outliers or noisy data. For these reasons it works well on collections of high affinity sites but its performance is degraded with any background or non-specific binding, and the quality of the model decreases rapidly as low quality, or even low affinity, data is added. BEEML doesn't suffer from those limitations because it models the complete distribution including non-specific binding so that the more data available the better it works, even if most of the sequences are non-specific. The algorithm is more complex and slower than QP, but still reasonably fast even for long sites when using the FFT to estimate the partition function.

Besides the introduction of BEEML, this paper also introduces a novel HT-SELEX procedure for accurate estimation of binding energies from in vitro selected sites. SELEX, and related methods, have been employed since 1990 to determine the specificity of DNA and RNA binding proteins, as well as for other purposes [34]–[36]. Most early uses of SELEX to determine binding specificities of TFs used multiple rounds of selection and amplification followed by cloning and sequencing of a small number of binding sites [3]. While this was sufficient to determine preferred sequences and the differential variability that is tolerated at different positions within the binding site, it generally did not provide very accurate models of binding specificity. Increasing the number of selected binding sites that were sequenced to several thousand, using a SAGE-SELEX method, improved the modeling accuracy [37]. But this approach still used multiple rounds of selection and amplification prior to sequencing, and with the prior probabilities, *P(S_i_)*, changing at every round, it is difficult to directly apply the biophysical model to that data. The QP approach has been applied to the analysis of the SAGE-SELEX data with some success [38],[39] but the same caveats remain for QP as described above. In our HT-SELEX approach we determine the prior sequence distribution directly, as well as the distribution of sequences in the bound fraction, so that we have all of the data necessary for the full model. And by collecting several hundred thousand sequences we have enough data to estimate the parameters of the model quite accurately even though the majority of the selected sites after one round are still non-specific, or at least low affinity. New sequencing methods, such as the Illumina approach, are ideal for this type of data. We only need short reads, 10bp randomized regions in the studies presented here but one could easily go to about 30bp randomized sequences and still obtain high quality data across the entire binding site. And with many millions of reads obtainable in a single lane, one can use multiplexing techniques to collect data for many different proteins, or many different conditions and constraints for the same protein, at once, reducing the cost per experiment.

Further developments of this approach are underway. While we show that a single round of HT-SELEX is sufficient to get reasonably accurate models of binding energy, we think that including data from additional rounds may provide even better models. Sequencing after each round means that we have good estimates for the prior sequence distributions at each round and since the energy model and non-specific energy will be the same, the only additional parameters to estimate are the chemical potentials at each round, 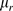. In succeeding rounds the prior distribution is already enriched with high affinity sites, so the posterior distributions should help us refine the model and ascertain more accurately the contributions of higher order combinations of bases.

Other types of data should also be amenable to the BEEML approach. We demonstrated its application to affinity data from MITOMI experiments [8],[28] and how it can help identify an appropriate binding model, including higher order contributions. Protein Binding Microarray (PBM) is an important new experimental approach that can provide very high throughput binding data [7]. The measurements are not affinities directly but are related to them and our approach, with some modifications, should be able to generate accurate models, including multiple modes of binding and higher order interaction contributions. HT-SELEX has one important advantage over PBM which is that it can analyze much longer binding sites, up the read length of the sequencing methods although beyond about 30bp the size of the available library becomes limiting. As many proteins, especially bacterial transcription factors, bind as dimers to sites of about 20bp or longer, HT-SELEX may be able to determine their specificities accurately when the PBM approach, which is currently limited to site sizes of about 10bp, would not. Another approach that is capable of determining specificity for very long binding sites is the bacterial-one-hybrid (B1H) approach [40]. It has an advantage that the binding proteins do not need to be purified, merely cloned and expressed in the bacterial cells. The library size is limited by the number of transformants one can obtain, which will be quite a bit smaller than *in vitro* libraries, but still large enough to sample very many potential binding sites. One complication arises in analyzing data from B1H experiments, and with any data about binding sites that have been selected for function *in vivo*, which is that their statistics are not determined solely by binding affinity, but also include selection constraints [41]. In particular, there may be a lower bound on affinity such that sites with lower affinity will not be occupied sufficiently well to survive selection. But additionally there may be no further selection for the highest affinity sites, all sites that “good enough” are equally likely to survive, and there could even be negative selection against sites that have affinities that are too high. Such constraints violate the fundamental premise of the biophysical model, but are consistent with the QP model and may explain why it works quite well on relatively small samples of known regulatory sites. But further modifications to our model that include selection constraints may lead to improved accuracies on a wide variety of data sources.

A current source of *in vivo* data that is growing rapidly is from ChIP-chip and ChIP-Seq experiments [42],[43] which are likely to have a mixture of the constraints described above. Some sites will be selected based primarily on their affinity, and there can be substantial background binding included, and additionally there will be sites surviving evolutionary selection that may have affinities optimized for function but not for highest affinity. In eukaryotic cells there are also the confounding effects of nucleosomes and the fact that many potential sites may not be accessible to the TFs, as well as cooperative effects of multiple TFs binding together in *cis*-regulatory modules. Such datasets also require a motif discovery, and perhaps alignment, step in order to identify the bound sites within the larger regions that are obtained in the experiments. We think that further development of the BEEML approach will increase its applicability to those types of data as well.
